# Asylum seekers need to be treated under civil rights laws in the USA

**DOI:** 10.1016/j.lana.2023.100578

**Published:** 2023-08-11

**Authors:** The Lancet Regional Health – Americas

The United Nations Universal Declaration of Human Rights states that, “everyone has the right to seek and to enjoy in other countries asylum from persecution”. Latin Americans from several countries (ie Mexico, Guatemala, Honduras) seek asylum in the USA, looking for protection, security, and a way to provide for their families. Those who seek asylum must provide evidence that they meet the definition of a refugee. Despite the declaration of Human Rights, the civil rights of immigrants in the USA have continued to be violated. The denouncement of an e-mail stating that migrant children were being pushed into the Rio Grande river by Texas troopers, a girl dying after being denied medical care, and the installation of a floating barrier by the state of Texas to stop people from swimming to cross the border at the Rio Grande river confirm that the violence against immigrants is far from over, once again crossing the line of basic human rights. Despite the termination of Title 42, the immigration policy implemented during President Trump’s administration that allowed the removal of migrants crossing the USA–Mexico border, President Biden’s restrictions on asylum seekers are still very strict and invite the question of whether his statement on humane treatment and civil rights for immigrants will actually be enforced.

At the beginning of the COVID-19 pandemic, Title 42 was implemented as a public health measure that allowed US authorities to expel migrants, including asylum seekers, at the US border. Title 42 was a controversial act ordered by the US Centers for Disease Control and Prevention, without scientific evidence supposed to protect people from being infected by COVID-19. As pointed out by Ulrich and Crosby, Title 42 was a justification to oppress marginalised populations, such as immigrants, those living in poverty, women, and people of colour, and denying access to human rights of immigrants and asylum seekers. More than 13,480 reports of violent conduct, including murder, torture, and kidnapping, have been reported since President Biden took office on Jan 20, 2021. Although COVID-19 cases dropped in most countries months before, Title 42 was only terminated on May 11, 2023, days after the declaration by WHO about the end of the global health emergency. With the termination of Title 42, President Biden proposed a rule to deny asylum to migrants at the USA–Mexico border, which was blocked by judge Jon Tigar from California.

The new restrictions present challenges for people seeking asylum, as those who enter the USA between legal border crossings are considered ineligible. Thus, people who do not request protection in transit countries, such as Mexico, or cross between ports of entry, are disqualified from claiming asylum and denied re-entrance to the USA for 5 years. Asylum seekers must wait in Mexico and are required to wait for their immigration court hearings that can last more than a year. Over the past 15 years, the number of border patrol arrests have increased greatly and, as discussed by Cameron and colleagues, families must stay in shelters that pose risks for their health due to overcrowding, poor sanitation, and precarious infrastructure. Violence against women has also been reported, with high rates of rape and feminicide by people involved in organised crime and by USA and Mexican officers. Children suffer with lack of education and family separation, which have huge impacts on their mental health and increasing risk for post-traumatic disorder. For the children that eventually cross the border, the Deferred Action for Childhood Arrivals programme (DACA), that provided work authorisation and deportation protection for those who entered in the USA as children is no longer an option. In October, 2022, the DACA programme was put on hold on the basis that it was unlawful, preventing new applications and creating speculation about its termination. The programme, implemented by President Obama in 2012, created new opportunities for more than 800,000 children since its start. Although not providing citizenship, one of its benefits was access to employer-sponsored and state-funded health insurance in certain states. One of President Trump’s main goals was to terminate DACA and, although this attempt was blocked by the Supreme Court, the decision to stop new applications has shown how the programme’s fragility. However, President Biden is trying to increase Medicaid and ACA health coverage to those under DACA. In a thoughtful and provocative viewpoint, Park and colleagues presented some possible paths for continuity of care of migrant population in the USA, such as state-level policymaking to expand coverage to all residents despite their migration status, and expanding health insurance eligibility for undocumented migrants.

As thousands of people arrive at the USA border requesting asylum, it is only fair that they are treated with the same dignity as any other person. They are vulnerable members of society that have suffered persecution and trauma and their rights must be guaranteed. With the termination of Title 42, the USA is in a position that could be decisive for anyone seeking to immigrate to the country. Immigrants are crucial components of American society and contribute to the economy and industry as, among other things, workers and tax payers. Undocumented immigrants do not increase violent crime rates, present lower incarceration rates than those born in the USA, and are less likely to be arrested for homicide. It is up to President Biden to decide if the USA will take a step in the right direction to guarantee proper rights for all those that seek asylum. Title 42 showed the consequences of major actions within the migrant population, having a huge impact on the mental health and long-term development of both adults and children. It is time now to reconcile and provide proper support and resources for migrants to the USA.

